# Beer? Over here! Examining attentional bias towards alcoholic and appetitive stimuli in a visual search eye-tracking task

**DOI:** 10.1007/s00213-019-05313-0

**Published:** 2019-07-08

**Authors:** Charlotte R. Pennington, Adam W. Qureshi, Rebecca L. Monk, Katie Greenwood, Derek Heim

**Affiliations:** 1 Department of Health and Social Sciences, Faculty of Health and Applied Sciences, University of the West of England, Coldharbour Lane, Bristol, BS16 1QY UK; 2 Department of Psychology, Edge Hill University, St Helens Road, Ormskirk, Lancashire L39 4QP UK

**Keywords:** Alcohol consumption, Attentional bias, Appetitive processing, Visual search, Eye-tracking

## Abstract

**Rationale:**

Experimental tasks that demonstrate alcohol-related attentional bias typically expose participants to single-stimulus targets (e.g. addiction Stroop, visual probe, anti-saccade task), which may not correspond fully with real-world contexts where alcoholic and non-alcoholic cues simultaneously compete for attention. Moreover, alcoholic stimuli are rarely matched to other *appetitive* non-alcoholic stimuli.

**Objectives:**

To address these limitations by utilising a conjunction search eye-tracking task and matched stimuli to examine alcohol-related attentional bias.

**Methods:**

Thirty social drinkers (*Mage =* 19.87, *SD* = 1.74) were asked to detect whether alcoholic (beer), non-alcoholic (water) or non-appetitive (detergent) targets were present or absent amongst a visual array of matching and non-matching distractors. Both behavioural response times and eye-movement dwell time were measured.

**Results:**

Social drinkers were significantly quicker to detect alcoholic and non-alcoholic appetitive targets relative to non-appetitive targets in an array of matching and mismatching distractors. Similarly, proportional dwell time was lower for both alcoholic and non-alcoholic appetitive distractors relative to non-appetitive distractors, suggesting that appetitive targets were relatively easier to detect.

**Conclusions:**

Social drinkers may exhibit generalised attentional bias towards alcoholic and non-alcoholic *appetitive* cues. This adds to emergent research suggesting that the mechanisms driving these individual’s attention towards alcoholic cues might ‘spill over’ to other appetitive cues, possibly due to associative learning.

**Electronic supplementary material:**

The online version of this article (10.1007/s00213-019-05313-0) contains supplementary material, which is available to authorized users.

## Introduction

Attentional bias (AB) is the tendency for an individual’s focus to be drawn to certain preferred cues, and a wealth of research suggests that this process may underpin addictive behaviours (see Field and Cox 2008 for a review). Studies have shown that both dependent (Cox et al. 2002) and non-dependent drinkers (Melaugh-McAteer et al. 2015) display AB towards alcohol-related stimuli, which appears to be proportionally determined by individual differences in consumption (Field et al. 2014). The process of AB has been explained by the incentive sensitisation theory of addiction (Berridge and Robinson 2016; Robinson and Berridge 1993, 2001). Here it is postulated that repeated consumption causes alcohol-related stimuli to acquire incentive-motivational properties, which potentiates attentional resources and encourages further use. In other words, being motivated to consume alcohol alters the way in which associated cues are perceived to influence attentional orienting (Field and Cox 2008; Field et al. 2014).

AB for alcohol-related cues can be measured through direct measures (e.g. eye movements) or inferred through indirect assessments (e.g. response time). Indirect measures such as the addiction Stroop task (see Cox et al. 2006) typically indicate that heavy social drinkers exhibit slower responses to alcoholic relative to neutral stimuli (e.g. Bruce and Jones 2004; Fadardi and Cox 2008; Field et al. 2007; Sharma et al. 2001; White et al. 2014). Likewise, heavy drinkers’ performance tends to be impaired towards alcoholic relative to neutral stimuli when individuals are exposed to an alcoholic beverage prior to completing this task (Cox et al. 2003). Other research using a diverse array of behavioural tasks, such as the flicker change blindness paradigm (Jones et al. 2002, 2006), attentional cueing (Garland et al. 2012; Stormark et al. 1997), dual processing (Waters and Green 2003), rapid serial visual presentation (Brown et al. 2018) and cued target detection tasks (Abroms and Fillmore 2004) also point to AB as an important mechanism shaping alcohol consumption behaviours.

Studies employing the visual probe task as an indirect measure of AB, however, have yielded somewhat mixed findings. Some research indicates that heavy social drinkers respond faster to visual probes that replace alcohol-related stimuli (Townsend and Duka 2001), which appears to influence subjective craving (Manchery et al. 2017; c.f., Field et al. 2010). Other research has shown, however, that this effect may only emerge when stimuli are presented for longer durations (i.e. 500–2000 ms vs. 200 ms; Field et al. 2004), and for those with lower effortful control (van Hemel-Ruiter et al. 2015). Recently, the visual probe task has been shown to suffer from low internal and test–retest reliability (Jones et al. 2018; see also Ataya et al. 2012; Field and Christiansen 2012), which may explain the heterogeneity of previous findings. It has therefore been suggested that researchers may obtain more reliable measures of AB by employing direct measures (Christiansen et al. 2015; Field and Cox 2008; Miller and Fillmore 2010).

Research examining the impact of alcohol cue exposure on oculomotor (eye movement) responses broadly replicates response patterns observed from indirect measures. For example, Melaugh-McAteer et al. (2015) found that adolescent social drinkers orient faster towards alcohol-related appetitive (relative to neutral) stimuli on the anti-saccade task. Moreover, drinkers have been shown to exhibit AB towards alcohol-related cues on the visual probe eye-tracking task (Fernie et al. 2012; Miller and Fillmore 2010). Similarly, Wilcockson and Pothos (2015) found that increased alcohol use was related to the reallocation of attention from central fixation towards peripheral alcoholic stimuli (termed ‘break frequency’). Research from both direct and indirect measures of AB therefore suggests that individuals who frequently consume alcohol might allocate attentional resources disproportionately towards alcohol-related stimuli.

Despite the convergence of these findings, questions can be raised with regard to the stimuli typically used in relevant experimental paradigms. Specifically, responses to alcohol-related stimuli (e.g. beer, wine, spirits) are usually contrasted with non-appetitive neutral stimuli (e.g. stationary and household objects; Bruce and Jones 2004; Fernie et al. 2012; Field et al. 2011; Jones et al. 2006; Townsend and Duka 2001; White et al. 2014; Wilcockson and Pothos 2015). It is possible, however, that comparing responses between appetitive (alcohol) and non-appetitive (control) stimuli may exaggerate perceptions of the extent to which AB is exhibited towards alcoholic cues (Field and Cox 2008). To wit, Monk et al. (2017) found what they referred to as a ‘spill over effect’ whereby participants exhibited diminished inhibitory control towards both alcoholic and non-alcoholic *appetitive* stimuli relative to non-appetitive stimuli. Similarly, Wiers et al. (2009) found that heavy drinkers displayed AB towards other appetitive stimuli (i.e. soft drinks as well as alcohol) and Qureshi et al.’ (2019) findings indicate that heavy social drinkers shift overt attention towards alcoholic and non-alcoholic appetitive stimuli. This emerging body of work therefore highlights the possibility that the mechanisms driving attention towards alcoholic cues might generalise to other appetitive non-alcoholic cues.

Previous research in this field may also be limited by its reliance on relatively simple target detection tasks, which tend to contrast single-stimulus targets. Tasks such as the anti-saccade and addiction Stroop task, for example instruct participants to respond to a single alcohol-related or neutral stimulus. The visual probe task typically presents two contrasting alcoholic and non-alcoholic images and requires participants to respond to a probe that replaces them. Nevertheless, these tasks are not representative of real-world environments in which drinkers are often exposed to numerous different alcoholic and non-alcoholic beverages that simultaneously compete for attention. Consequently, it is not fully known whether social drinkers display AB towards alcoholic stimuli embedded in an array of other non-alcoholic and non-appetitive products. Indeed, visual attention is most often engaged in settings that involve multiple objects including both relevant targets and irrelevant distractors. As such, the use of more sophisticated visual search paradigms may be warranted to fully elucidate the nature of alcohol-related AB.

In existing visual search tasks, participants indicate whether a predefined target is present or absent within a larger array of multiple distractors. According to the feature integration theory (Treisman and Gelade 1980; Treisman and Souther 1985), parallel search is adopted when a target is characterised by a single feature (e.g. colour) that is not shared with the distractors because the target appears to ‘pop out’. Conversely, serial search processes are necessary to identify targets characterised by a conjunction of features shared by the distractor items (e.g. colour, size, orientation). Serial search processing demands more allocation of attentional resources compared with bottom-up, parallel search processing. Consequently, searches made under these conditions are comparatively slower and increase in a linear fashion with the addition of distractors (Eckstein et al. 2000; Narbutas et al. 2017; Treisman and Gelade 1980). Because of the low feature contrast between the target and distracter elements, participants must use knowledge of the specific features that characterise the target to guide their searches. As such, conjunction search tasks may be ideal for assessing alcohol-related AB because through repeat exposure and use, individuals who consume alcohol may attentionally prioritise the detection of alcoholic cues in their environment. In line with this assertion, prior work indicates that attentional resources can be influenced by stimuli imbued with value via associative learning (Anderson et al. 2011).

Some studies have examined the psychopharmacological effects of alcohol administration on visual search (e.g. Abroms and Fillmore 2004; Hoyer et al. 2007; Maylor et al. 1987; Moskowitz et al. 1976; see Olthuis and Klein 2012 for a review). Findings indicate that acute alcoholic intoxication impairs visual search performance by decreasing accuracy and increasing response time. To date, however, only one study (Brown et al. 2018) has used an adaptation of a visual search paradigm to examine alcohol-related AB in non-intoxicated individuals. Here, participants were given search goals to detect an alcoholic (e.g. beer) or non-alcoholic target (e.g. a shoe) when presented with unrelated, everyday objects. Prior to viewing potential targets, a task-irrelevant alcoholic or non-alcoholic distractor appeared in parafoveal locations which participants were instructed to ignore. Findings across three experiments indicate that when participants held a search goal for alcohol-related targets, there was consistent AB to task-irrelevant alcoholic but not to non-alcoholic distractors. Brown et al. suggest that social drinkers may be attuned to alcohol in their environment, resulting in involuntary contingent capture by alcoholic stimuli. Nevertheless, like other research in this area, this study compared the detection of alcoholic stimuli with non-matched, non-appetitive stimuli (e.g. household objects) and inferred alcohol-related AB through an indirect behavioural measure (i.e. key presses). Expanding upon this, the current study utilises a conjunction search eye-tracking task to assess directly whether social drinkers demonstrate alcohol-related AB relative to both non-alcoholic appetitive and non-appetitive stimuli.

### Overview of current research

The current study takes a more ecological approach to assessing alcohol-related AB in social drinkers, whilst also considering the effect of (non)appetitive cues used within such tasks. To achieve this, we employed a conjunction search task—a mainstream cognitive test that has to date not been widely deployed in alcohol research—to assess alcohol-related AB. Participants were instructed to search for an alcoholic (beer), non-alcoholic appetitive (water) or non-appetitive (detergent) target amongst an array of other matching and non-matching distractors. Both behavioural response times (i.e. RT to indicate whether the target was present/absent) and proportional dwell time (i.e. eye movements indicative of the time spent fixating on distractors matching the target) were measured. It was predicted that if social drinkers demonstrate AB towards alcohol solely then they would be quicker to detect alcoholic targets (beer) compared to both non-alcoholic appetitive (water) and non-appetitive (detergent) targets. Similarly, proportional dwell time on matching distractors was hypothesised to be lower on trials in which the target was alcoholic because these should be detected with relative ease. Conversely, as demonstrated in emergent research (see Monk et al. 2017; Qureshi et al. 2019), if AB spills over to other appetitive stimuli, then participants would be quicker to detect both alcoholic and non-alcoholic appetitive targets (i.e. beer and water) compared with non-appetitive targets (detergent). Further, proportional dwell time on both alcoholic and non-alcoholic matching distractors would be lower relative to non-appetitive matching distractors.

## Method

### Participants and design

Participants completed a visual search task comprising a 3 (visual target: alcohol appetitive, non-alcohol appetitive, non-alcohol non-appetitive) × 2 (array size: small [24], large [36]) × 2 (target presence: present vs. absent) within-participants design. Based on our analytical procedure, power analyses (G*Power; Faul et al. 2007) indicated that a sample size of 34 participants was required to detect a moderate effect size (Cohen’s *f* = .25) for main effects with 80% power. A total of 45 participants signed up to the study through an online server; however, 15 were excluded due to unmatched memorable dates between the online pre-test questionnaire and the eye-tracking task (*n* = 11), duplicate questionnaire responses (*n* = 1) or not showing up to the experimental testing phase (*n* = 3). The final sample therefore consisted of 30 participants (19 female; 80% White British) between the age of 18 and 25 (*M*age = 19.87, *SD* = 1.74), all of whom reported normal or corrected-to-normal visual acuity.

### Measures

#### Conjunction search task

Participants completed a conjunction search task (Treisman and Sato 1990; Treisman and Souther 1985) programmed in Experiment Builder (SR Research Ltd. 2017). Eye movements were measured throughout using a video-based pupil-tracking system (EyeLink 1000, SR Research Ltd.) with a sampling rate of 250 Hz. To measure behavioural responses, participants were instructed to identify whether a given visual target was ‘present’ or ‘absent’ by pressing the green and red buttons, respectively, on an SR Research gamepad.

Search arrays consisted of appetitive alcohol (beer bottle), non-alcohol (water bottle) and non-appetitive (detergent) coloured stimuli displayed randomly and equally in set sizes of 24 and 36. These stimuli were validated in a previous study by Monk et al. (2017) whereby participants had to identify whether the stimuli shown were appetitive or non-appetitive. Unbranded products were chosen due to concerns that brand influence could unduly affect attentional bias (see Domaradzka and Bielecki 2017). To allay fears regarding the luminosity of the different stimuli (i.e. beer–water–detergent bottles; see Frey et al. 2008), we also administered a greyscale version of the task, which was counterbalanced between participants. The findings from the greyscale version are similar to the colour version and are reported in Supplementary File 1.^1^ We choose to focus on the coloured stimuli here as this is arguably more ecologically valid (i.e. the stimuli used are similar to that in real-world drinking environments).

**Fig. 1:**
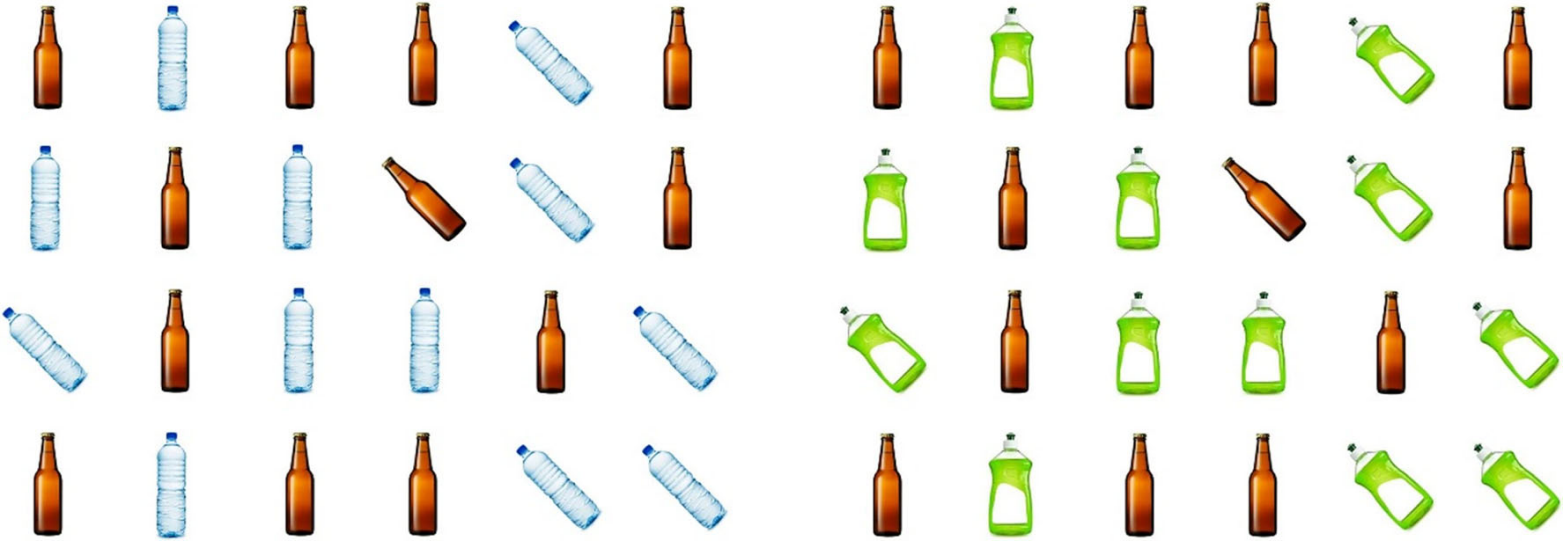
Example trial types. In these trials, participants were instructed to identify whether a left-hanging beer bottle was present or absent in an array of other non-alcoholic appetitive (left) and non-appetitive distractors (right)

The colour version of the visual search task comprised three critical blocks of 40 trials (*n* = 120 trials total), with six-trial types presented randomly throughout these blocks for a total of 20 trials. Participants completed five practice trials before completing these critical blocks, which were removed from final analyses. Each trial began with a prompt to continue, after which an image of the target was presented on-screen for 200 ms, followed by a fixation cross presented for 200–500 ms. The stimulus array was then presented until the participant made a response. There was a 200 ms inter-trial interval. Target stimuli were located amongst other matching and non-matching distractor items, with only two contrasting stimulus items used per trial (e.g. an alcoholic target located amongst matching alcoholic and non-matching non-alcoholic distractor items; see Fig. 1). This resulted in a total of six different trial-types, displayed in Table 1. Stimulus items subtended 1.4° of visual angle horizontally and 1.8 vertically at a viewing distance of 57 cm. The target stimuli were rotated 45° to the left and were present on 50% of randomised trials. There were two main dependent variables of interest. The first was behavioural response times (RT) for correct responses to detect whether the target was present or absent. Average response times were computed for each of the six-trial types. The second was proportional dwell time which is the percentage of time participants spent fixating on distractors that matched the target within the array (represented as a proportion of total RT for each trial). These were summed for each target and distractor type (e.g. time spent fixating on matching alcohol distractors when the target was also alcohol and the mismatching distractor was non-alcohol). Accuracy was > 98% and therefore not analysed.

**Table 1:** Trial types in the conjunction search task. Targets were present on 50% of randomised trials and array size varied equally and randomly between 24 and 36

Trial type	Trials	Target	Distractor
1	20	Alcohol appetitive (beer)	Non-alcohol appetitive (water)
2	20	Alcohol appetitive (beer)	Non-alcohol non-appetitive (detergent)
3	20	Non-alcohol appetitive (water)	Alcohol appetitive (beer)
4	20	Non-alcohol appetitive (water)	Non-alcohol non-appetitive (detergent)
5	20	Non-alcohol non-appetitive (detergent)	Alcohol appetitive (beer)
6	20	Non-alcohol non-appetitive (detergent)	Non-alcohol appetitive (water)

Internal reliabilities for each trial type are presented in Table 2 for behavioural RT and proportional dwell times. The reliability of behavioural RT for the visual search task is considerably better and more stable than that reported for the visual probe task (*α* = .00–.50; mean = .18; Ataya et al. 2012; see also Christiansen et al. 2015) and is comparable to the addiction Stroop task for both RT (*a* = .53) and eye movement measures (*a* = .71; Field and Christiansen 2012). Consistent with findings from Christiansen et al. (2015), eye movement measures as a direct measure of attentional bias were more reliable than indirect reaction time measures.

**Table 2:** Internal consistencies (Cronbach’s *α*) for reaction time (RT) and proportional dwell time (PDT) on the visual conjunction search task

	Target presence	
	Present	Absent
Target type		
Alcoholic vs. non-alcoholic RT	.35	.86
Alcoholic vs. non-appetitive RT	.58	.82
Non-alcoholic vs. non-appetitive RT	.37	.83
Mean reliability for RT	.43	.84
Alcoholic vs. non-alcoholic PDT	.60	.66
Alcoholic vs. non-appetitive PDT	.55	.84
Non-alcoholic vs. non-appetitive PDT	.77	.79
Mean reliability for PDT	.64	.76

#### Alcohol use disorders identification test

The AUDIT (Saunders et al. 1993) was employed to measure participants’ harmful drinking behaviour. This questionnaire resulted in acceptable internal consistency (Cronbach’s *a* = .77) and a total score was computed. Scores of 8 or more are indicative of harmful drinking patterns and our sample generally consisted of social drinkers (*M =* 6.53, *SD* = 4.64).

#### Adult temperament questionnaire

The effortful control sub-scale of the ATQ (Rothbart et al. 2000) measured trait effortful control (EC). This 35-item questionnaire measures sub-components of attentional control, inhibitory control and activation control. Responses were recorded on a 7-point Likert scale (1, extremely untrue of you; 7, extremely true of you). This measure resulted in excellent internal consistency (Cronbach’s *a* = .90) and a mean score was calculated across the three sub-components (*M* = 3.70, *SD* = .71).

### Procedure

Ethical approval was granted from the institutional governing body and participants provided informed consent prior to taking part. Data collection was conducted solely at one testing site and was carried out in two phases: first, participants completed the AUDIT and ATQ questionnaires online to control for alcohol-related priming in the experimental phase (see Melaugh-McAteer et al. 2015). Second, participants arrived at the lab and provided the researcher with their memorable date to match questionnaire responses with their experimental data. Each participant was asked to place their head in a chin-rest situated 57 cm from the computer screen and their eye movements were calibrated using a 9-point tracking system. Before each trial, participants were shown a picture of the target they should search for and were instructed to press the green and red gamepad button for present and absent targets, respectively. They then completed the colour and greyscale version of the visual search task in counterbalanced order. For the coloured version reported here, participants completed a total of three blocks of 40 trials (*n* = 120 total), with breaks provided between each block to reduce fatigue. After completion of the experiment, participants received a written debrief, which explained the experimental aims and included contact numbers for alcohol-related support services.

## Results

A series of 2 (target type = e.g. alcoholic v. non-alcoholic) × 2 (target presence: present vs. absent) × 2 (array size: small, large) repeated measures ANOVAs were conducted to examine RTs to detect the target. Within these analyses, it was necessary to hold the distractor constant because sometimes the same distractor was used within a different trial type (e.g. alcoholic target vs. non-appetitive distractor, non-alcoholic target vs. non-appetitive distractor). The same analyses were then conducted on proportional dwell time (i.e. time spent fixating on distractors that matched the target). Here, areas of interest (AOI) were created for each stimulus in the search array (target and distractor). The time spent by participants gazing in various AOI was automatically calculated by Experiment Builder through the Data Viewer. A series of ANCOVAs were then conducted to examine whether alcohol consumption (AUDIT scores) or trait EC explained any variance in search performance. This was based on research demonstrating that problematic alcohol consumption is correlated positively with AB towards alcoholic stimuli (Field et al. 2010), whereas higher levels of EC allow individuals to override such prepotent responding (Morales et al. 2016; Posner et al. 2014; Qureshi et al. 2017; van Hemel-Ruiter et al. 2015). All main effects and interactions for both the ANOVA and ANCOVA analyses were elucidated using Bonferroni-corrected pairwise comparisons to control for type 1 error. Outliers above or below 2.5 SDs from the condition mean were removed.

### Behavioural RT

#### Alcoholic vs. non-alcoholic target (distractor = non-appetitive)

There was a significant main effect of target presence, with faster responses when the target was present (*M* = 1034.45, *SE* = 41.29) compared with absent (*M* = 1720.21, *SE* = 132.75), *F*(1, 27) = 37.65, *p* < .001, η_p_^2^ = .58. There was also a significant main effect of array size, with faster responses to the small (*M* = 1192.25, *SE* = 55.85) compared with large array (*M* = 1562.41, *SE* = 119.93), *F*(1, 27) = 15.50, *p* = .001, η_p_^2^ = .37. Of focal interest, there was no significant main effect of target type on RT (*p* = .75, η_p_^2^ = .004), and no significant interactions (all *p* > .05). Adding AUDIT as a covariate did not influence these results. Adding EC as a covariate removed the main effect of target presence and array size, suggesting that EC may account for some variance in search performance.

#### Alcoholic vs. non-appetitive target (distractor = non-alcoholic)

There was a significant main effect of target presence, with faster responses when the target was present (*M* = 985.09, *SE* = 38.75) compared with absent (*M* = 1624.19, *SE* = 92.43), *F*(1, 27) = 68.26, *p* < .001, η_p_^2^ = .72. There was also a significant main effect of array size, with faster responses to the small (*M* = 1124.20, *SE* = 51.95) compared with large array (*M* = 1485.08, *SE* = 72.79), *F*(1, 27) = 68.99, *p* < .001, η_p_^2^ = .72. Of focal interest, there was a significant main effect of target type, with faster responses to alcoholic (*M* = 1247.02, *SE* = 58.21) compared with non-appetitive targets (*M* = 1362.26, *SE* = 63.63), *F*(1, 27) = 17.28, *p* < .001, η_p_^2^ = .39. All interactions were non-significant, *p* > .05.

**Fig. 2:**
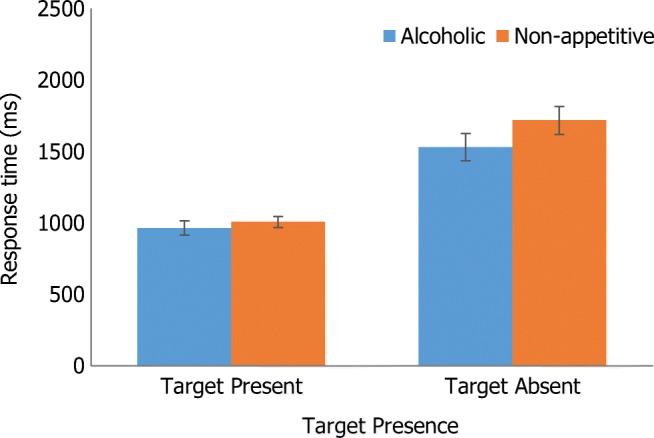
Two-way interaction between target presence and target type with AUDIT as a covariate. Error bars = standard error

Adding AUDIT as a covariate resulted in a significant two-way interaction between target presence and target type, *F*(1, 26) = 4.76, *p* = .038, η_p_^2^ = .16. Simple main effects indicated that RT was quicker when the target was present compared with absent for both alcoholic and non-appetitive targets (all *p* < .001). When the target was absent, participants were faster to detect the alcoholic relative to the non-appetitive target (*p* < .001), but there was no difference when the target was present. Figure 2 displays this interaction. Adding EC as a covariate removed the main effects of target presence and target type, but the main effect of array size remained, though with reduced effect size, *F*(1, 26) = 5.66, *p* = .025, η_p_^2^ = .18.

#### Non-alcoholic vs. non-appetitive target (distractor = alcoholic)

**Fig. 3:**
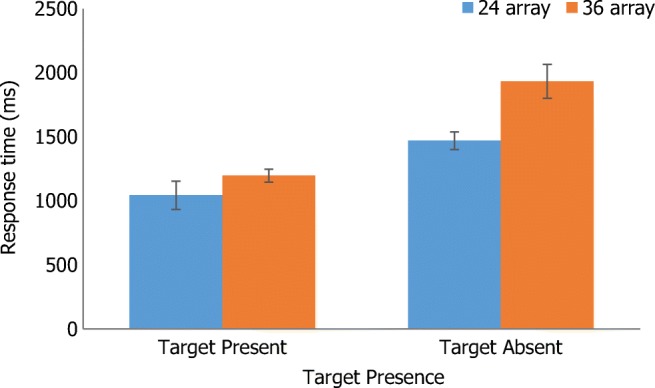
Two-way interaction between target presence and array size. Error bars = standard error

There was a significant main effect of target presence, with faster responses when the target was present (*M* = 1119.60, *SE* = 66.11) compared with absent (*M* = 1699.20, *SE* = 95.99), *F*(1, 28) = 39.93, *p* < .001, η_p_^2^ = .59. There was a significant main effect of array size, with faster responses to the small (*M* = 1255.47, *SE* = 69.73) compared with large array (*M* = 1563.33, *SE* = 84.45), *F*(1, 28) = 18.11, *p* < .001, η_p_^2^ = .39. Of focal interest, there was a significant main effect of target type, with faster responses to non-alcoholic (*M* = 1307.50, *SE* = 61.88) compared with non-appetitive targets (*M* = 1511.30, *SE* = 86.70), *F*(1, 28) = 10.55, *p* < .01, η_p_^2^ = .27. There was also a significant two-way interaction between target presence and array size, *F*(1, 28) = 4.78, *p* < .05, η_p_^2^ = .15. Simple main effects indicated that participants were faster when the target was present compared with absent regardless of array size (all *p* < .01). Participants were faster to respond to the small array compared with large array when the target was absent (*p* < .01), but there was no significant difference when the target was present (*p* = .18). Figure 3 displays this interaction.

**Fig. 4:**
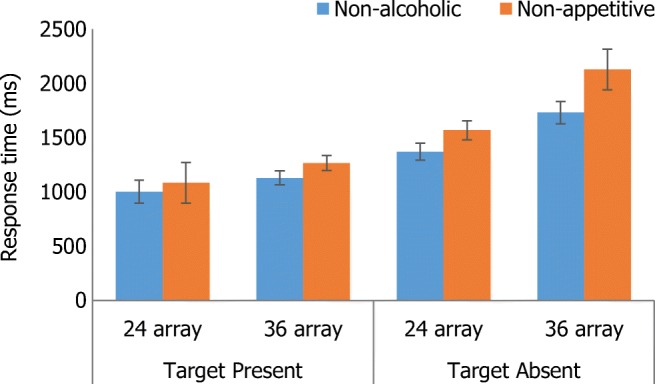
Three-way interaction between target presence, array size and target type with EC as a covariate

Adding AUDIT as a covariate removed this interaction between target presence and array size, but the main effects of target presence, array size and target type remained. Including EC as a covariate removed the main effects and interactions present in the ANOVA, but revealed a three-way interaction between target presence, array size and target type, *F*(1, 27) = 6.29, *p* < .05, η_p_^2^ = .19. Simple main effects indicated that RT was faster when both the non-alcoholic and non-appetitive target was present compared with absent in both the small and large array (all *p* < .001). When the target was absent, RT for both the non-alcoholic and non-appetitive target was slower for the large array compared with the small array (all *p* < .01) but participants were faster to respond that a non-alcoholic target was absent compared with when the target was non-appetitive (all *p* < .05). Figure 4 displays this interaction.

### Proportional dwell time

#### Alcoholic vs. non-alcoholic matching distractors (non-matching distractor = non-appetitive)

There was a significant main effect of target presence, with shorter proportional dwell time on matching distractors when the target was absent (*M* = .27, *SE* = .02) compared with present (*M* = .36, *SE* = .01), *F*(1, 28) = 21.56, *p* < .001, η_p_^2^ = .44. There was a significant main effect of array size, with shorter proportional dwell time for the small (*M* = .27, *SE* = .02) compared with large array (*M* = .36, *SE* = .02), *F*(1, 28) = 14.73, *p* = .001, η_p_^2^ = .35. There was no significant main effect of distractor type (i.e. alcoholic vs. non-alcoholic, *p* = .55, η_p_^2^ = .01), and no significant interactions (all *p* > .05). Adding AUDIT as a covariate did not affect the results, though adding EC as a covariate removed the main effects of target presence and array size.

#### Alcoholic vs. non-appetitive matching distractors (non-matching distractor = non-alcoholic)

**Fig. 5:**
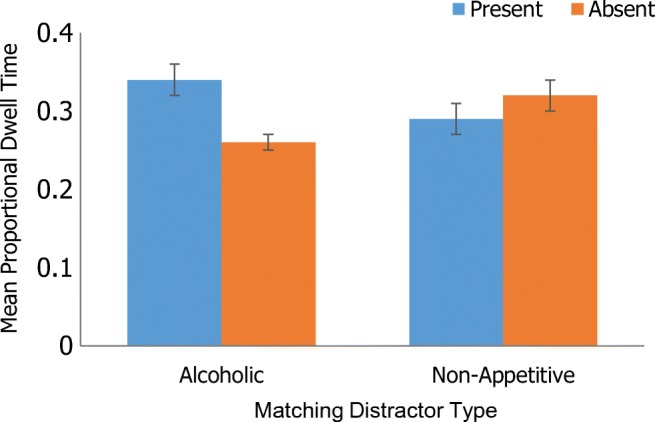
Two-way interaction between distractor type and target presence

There was a significant main effect of array size, with shorter proportional dwell time for the small (*M* = .25, *SE* = .02) compared with large array (*M* = .36, *SE* = .01), *F*(1, 28) = 46.49, *p* < .001, η_p_^2^ = .62. There was no significant main effect of target presence (*p* = .10, η_p_^2^ = .10) or distractor type (*p* = .45, η_p_^2^ = .02). There was, however, a significant two-way interaction between distractor type and target presence, *F*(1, 28) = 11.83, *p* < .01, η_p_^2^ = .30. When the target was absent, proportional dwell time was significantly shorter for alcoholic (*M* = .26, *SE* = .01) relative to non-appetitive distractors (*M* = .32, *SE* = .02), *p* = .01. There was no significant difference when the target was present (*p* = .07). Moreover, dwell time was shorter for matching alcoholic distractors when the target was absent (*M* = .26, *SE* = .01) compared with present (*M =* .34, *SE* = .02), *p* < .001. Dwell time on matching non-appetitive distractors did not significantly differ as a function of target presence (*p* = .36). Figure 5 displays this interaction. Adding AUDIT as a covariate did not significantly affect the results, though adding EC as a covariate removed all significant effects.

#### Non-alcoholic vs. non-appetitive matching distractor target (non-matching distractor = alcoholic)

**Fig. 6:**
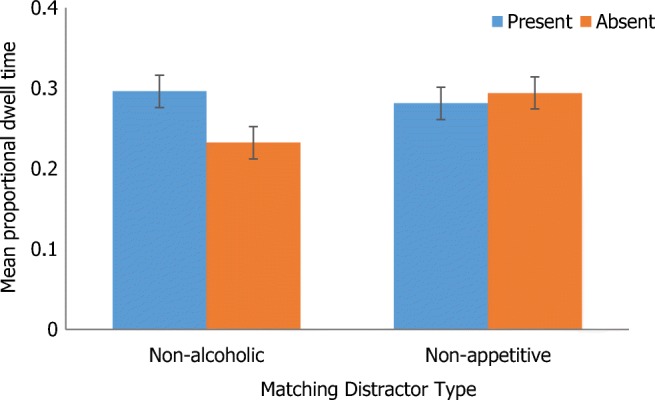
Two-way interaction between distractor type and target presence

There was a significant main effect of array size, with lower proportional dwell time for the small (*M* = .20, *SE* = .01) compared with large array (*M* = .35, *SE* = .02), *F*(1, 27) = 69.66, *p* < .001, η_p_^2^ = .72. There was no significant main effect of distractor type (*p* = .07, η_p_^2^ = .12) or target presence (*p* = .32, η_p_^2^ = .04). There was, however, two-way interaction between distractor type and target presence, *F*(1, 27) = 4.64, *p* = .04, η_p_^2^ = .15. Simple main effects showed that proportional dwell time was significantly lower for non-alcoholic (*M* = .23, *SE* = .02) compared with non-appetitive distractors (*M =* .29, *SE =* .02) when the target was absent (*p* < .01), but there was no significant difference when the target was present (*p* = .52). Figure 6 displays this interaction. All other pairwise comparisons were non-significant, *p* > .05.

Adding AUDIT as a covariate removed the two-way interaction between target presence and distractor type, but resulted in a significant main effect of distractor type, with lower proportional dwell time on non-alcoholic relative to non-appetitive distractors, *F*(1, 26) = 5.31, *p* < .05, η_p_^2^ = .17. Adding EC as a covariate resulted in a two-way interaction between array size and distractor type (*F*(1, 27) = 7.26, *p* < .05, η_p_^2^ = .22). Simple main effects showed that proportional dwell time was longer for both non-alcoholic and non-appetitive distractors for the large relative to the small array (*p* < .01). There were no other significant main effects or interactions (all *p* > .05).

## Discussion

The current study utilised a more ecological approach to assess alcohol-related AB in social drinkers, whilst also considering the effect of diverse (non)appetitive cues used within such tasks. Specifically, we used a conjunction search task to examine whether social drinkers demonstrate AB towards alcohol embedded within an array of multiple appetitive and non-appetitive cues. In line with the interpretations of prior research findings in this area, it was hypothesised that social drinkers demonstrating alcohol-related AB should exhibit quicker responses when detecting alcoholic relative to both non-alcoholic and non-appetitive targets. Similarly, they should also demonstrate lower proportional dwell time, detecting these targets with relative ease. In line with emergent research (Monk et al. 2017; Qureshi et al. 2019), however, it was hypothesised that if AB generalises to other appetitive stimuli, participants should exhibit faster responses and lower dwell time to both alcoholic and non-alcoholic *appetitive* targets relative to non-appetitive targets.

Current findings indicate that social drinkers were quicker to detect both alcoholic and non-alcoholic targets compared with non-appetitive targets. Qualifying this, there was no significant difference in search performance between alcoholic and non-alcoholic targets. At first glance, these findings seem to contrast with those reported by Brown et al. (2018) who found that social drinkers demonstrate AB towards alcoholic but not non-alcoholic distractors. Nevertheless, their study focused on the reallocation of attention to distractors, whereas the current study explored the detection of targets. Further, like many others in the literature, Brown et al. compared the detection of alcoholic stimuli with non-matched, non-appetitive stimuli (e.g. pots and pans). Instead, our findings suggest that AB towards alcoholic stimuli may generalise to other appetitive cues.

This assertion of a ‘spill over’ effect is consistent with research indicating that both social and heavy drinkers exhibit automatic approach tendencies towards alcoholic and non-alcoholic appetitive cues (see Monk et al. 2017; Qureshi et al. 2019; Wiers et al. 2009). Indeed, it has been theorised that the salience of appetitive cues may activate a general motivational state that enhances attention compared with low-incentive, non-appetitive cues (Monk et al. 2017; Wadhwa et al. 2008, see also Volkow et al. 2008, 2013). Neuroimaging research adds some weight to this assertion, with alcohol users showing activation in posterior brain regions that have been linked with appetitive functioning when viewing both alcoholic and non-alcoholic beverages (Tapert et al. 2003). In light of the current findings, it is therefore prudent for research in this field to utilise matched appetitive cues when examining alcohol-related cognitions. If stimuli are not matched then differential responding to alcoholic stimuli cannot be unequivocally attributed to its ‘substance-relatedness’ (Field and Cox 2008).

In the main, proportional dwell time findings mirrored those of behavioural response times. Fixations were lower for matching alcoholic relative to non-appetitive distractors when the target was absent amongst an array of mismatching non-alcoholic distractors. They were also lower when the alcoholic target was absent compared with present. This suggests that social drinkers found it easier, and were therefore quicker, to identify that an alcoholic target was absent in the array. Dwell time was also significantly lower on matching non-alcoholic relative to non-appetitive distractors when the target was absent amongst an array of mismatching alcoholic distractors. Revealing a general appetitive effect, there was no significant difference in the proportion of time spent fixating on alcoholic and non-alcoholic matching distractors in an array of non-appetitive mismatching distractors. Overall, these findings suggest that social drinkers may find it comparatively easier to detect both alcoholic and non-alcoholic appetitive targets when these are contrasted with non-appetitive distractors, perhaps because these stimuli are imbued with incentive value (see Monk et al. 2017). Complementing previous research using indirect behavioural measures, the current research may therefore provide a more nuanced insight into unconscious AB processes (Jonides 1981; Mogg et al. 2003; Wilcockson and Pothos 2015), suggesting further that AB towards alcoholic stimuli may generalise to other palatable non-alcoholic stimuli.

The current research also included AUDIT and trait EC scores as covariates in light of research suggesting that individual differences in problem drinking and the inhibition of prepotent responding are related to alcohol-related AB (see Morales et al. 2016; Posner et al. 2014; Qureshi et al. 2017; van Hemel-Ruiter et al. 2015). When comparing the detection of alcoholic relative to non-alcoholic targets, the addition of EC removed the main effects of target presence and array size for both RT and proportional dwell time. Indeed, research on conjunction search indicates consistently that RT is slower for absent targets and increases in a linear fashion with the addition of distractors (Eckstein et al. 2000; Narbutas et al. 2017; Treisman and Gelade 1980). The current finding may suggest that EC may account for variation in response and fixation duration; in other words, those with higher EC may demonstrate better search performance irrespective of task demands. Similarly, when comparing the alcoholic and non-appetitive conditions, the addition of EC removed the main effects of target type for behavioural RT. This may suggest that the ability to override prepotent responding allows social drinkers to inhibit typically quicker responses towards alcoholic targets.

Including AUDIT as a covariate resulted in a two-way interaction between target presence and target type for the alcoholic relative to non-appetitive target condition. Equivalent to the main analyses, findings indicate that participants were significantly quicker to identify that an alcoholic relative to a non-appetitive target was absent in the array. However, the inclusion of AUDIT scores removed the primary finding that responses towards alcoholic targets were faster when the target was present. This suggests that self-reported alcohol consumption may explain some variance in alcohol-related AB, with social drinkers being quicker to detect the presence of alcoholic targets when typical consumption behaviours are not accounted for. Such interpretations are, however, speculative and future research is recommended to examine whether high and low drinkers show different patterns on this task.

### Limitations and future directions

It is important to acknowledge that the current study utilised a conjunction search task, whereby two features distinguish the target from the distractors (i.e. colour, orientation) compared with parallel search whereby the target is distinguished by a single feature. In contrast to parallel search, conjunction search is theorised to involve consciously controlled, top-down processing, and there is debate as to whether automatic or controlled processing underpins alcohol-related AB (see Ceballos et al. 2009; Melaugh-McAteer et al. 2015). For example, Melaugh-McAteer et al. (2015) suggest that alcohol AB in social drinkers may be underpinned by controlled attention whereas automatic processing may develop in heavier drinkers. As such, it is possible that divergent results would emerge when using this task with heavy drinkers. Future research could assess this by employing both conjunction and parallel search tasks to assess whether performance differs between light and heavy drinkers and is underpinned by distinct processes. From this perspective, it is also worth noting that whilst automatic orienting may be characteristic of alcohol dependence, the preferential attention shown towards alcohol for social drinkers may be a consequence of familiarity and not an indicator of misuse (Melaugh-McAteer et al. 2015).

The visual search task had arguably better ecological validity than prior tasks that present single-stimulus images (e.g. Stroop, visual probe, anti-saccade). However, it must be noted that the images used here were somewhat simplistic and may not fully capture alcohol-related attentional bias in the real world. Specifically, alcoholic and non-alcoholic images were presented against a blank background and consequently our design does not acknowledge drinking context as an important and increasingly recognised driver of alcohol-related cognitions (see Heim and Monk 2017; Monk and Heim 2013a, b, 2014; Thrul et al. 2017; Pennington et al. in press)*.* Furthermore, we selected only one type of beverage (i.e. beer, water, detergent) and removed branding to ensure that the visual characteristics of the drink did not unduly influence attentional bias (see Domaradzka and Bielecki 2017). Nevertheless, this means that people’s personal drinking preferences were not accounted for, which may influence attentional bias further (see Christiansen et al. 2015). Future research may therefore benefit from the inclusion of alcohol- and non-alcohol-related scenes, personalised stimuli and branding.

Finally, one strength of our experimental design was that participants completed a measure of self-reported alcohol consumption (AUDIT) and trait effortful control (ATQ) online prior to the experimental testing phase. This was to ensure that participants were not primed by the alcohol-related questionnaire content (see Melaugh-McAteer et al. 2015). However, our final sample size (*n* = 30) was slightly below target (*n =* 34) owing to a large proportion of mismatched identifiers between the pre-test questionnaire and eye-tracking task. Sensitivity power analyses suggest that our final sample size was adequately powered to detect moderate-large main effects (Cohen’s *f* > .25, 80% power), but only large interaction effects. Future studies are therefore warranted to replicate and extend these findings, as well as examining the utility of visual search paradigms in the investigation of alcohol-related attentional bias.

## Conclusion

The current study utilised a conjunction search paradigm and matched stimuli to examine whether social drinkers exhibit AB towards alcohol or whether this effect generalises across *appetitive* stimuli. Findings indicate that participants were quicker to detect the presence of alcoholic and non-alcoholic appetitive targets relative to non-appetitive targets. Similarly, they appeared to fixate on these targets for a shorter duration, suggesting they detected them with relative ease. These findings support emergent research (Monk et al. 2017; Qureshi et al. 2019) suggesting that social drinkers may exhibit generalised AB towards appetitive stimuli. Indeed, past research in this area predominantly contrasts responses to alcohol-related targets with non-matched control targets (e.g. office and household objects; Brown et al. 2018; Bruce and Jones 2004; Fernie et al. 2012; Field et al. 2011; Jones et al. 2006; Townsend and Duka 2001; White et al. 2014; Wilcockson and Pothos 2015). Given that appetitive processing is theorised to underlie addictive behaviours, it may be argued that utilising other appetitive stimuli may mask the detection of alcohol-related AB. Nevertheless, by not controlling for the appetitive nature of such stimuli in prior work, the effects of alcoholic stimuli on attentional biases may have been overstated.

## Electronic supplementary material

ESM 1 (DOCX 18 kb)

## Footnotes

1 There were some notable differences between responses to the coloured and greyscale version of the task. In the greyscale version, people were quicker to detect non-alcoholic over alcoholic stimuli, but there was no significant difference between these in the coloured version. Furthermore, dwell time was lower for alcoholic relative to non-appetitive distractors when the target was absent in the coloured version; however, this effect did not emerge when stimuli were presented in greyscale. This may suggest that stimuli features such as colour and luminosity have an effect on the attentional mechanisms we are aiming to study.
