# Supplementary material for: Beer? Over here! Examining attentional bias towards alcoholic and appetitive stimuli in a visual search eye-tracking task
Source: Psychopharmacology (Berl). 2019 Jul 8;236(12):3465–76. doi: 10.1007/s00213-019-05313-0 (PMC6892770; doi:10.1007/s00213-019-05313-0)
Supplement: Supplementary file 1 — (DOCX 18 kb) [file 213_2019_5313_MOESM1_ESM.docx]

**Supplementary Information File 1**

**Greyscale Version of Visual Search Task**

Due to concerns over the luminosity of stimuli (e.g., brightness of beer, water, detergent bottles) unduly affecting attentional bias, we also ran a greyscale version of the same task to corroborate our findings, which we counterbalanced between participants. Findings from this task were very similar, although in the greyscale version there was a significant main effect of target type between the alcoholic and non-alcoholic targets that was not present in the colour version (see below). The results pertaining to our focal analyses of target type (and associated interactions) for the greyscale version are presented here.

**Behavioural RT**

*Alcoholic v Non-Alcoholic target (Distractor = Non-appetitive)*

In contrast to the colour version of the task, there was a significant main effect of target type in the greyscale version, *F*(1, 29) = 5.98, *p* = .021, η_p_^2^ = .17. Here, participants responded faster to non-alcoholic targets (*M* = 1602.57, *SE* = 71.11) compared to alcoholic targets (*M* = 1774.11, *SE* = 112.43), *p* = .021.

*Alcoholic v Non-appetitive target (Distractor = Non-alcoholic)*

Consistent with the colour version, a significant main effect of target type was observed in the greyscale version, with faster responses to alcoholic targets (*M* = 1511.23, *SE* = 69.76) compared to non-appetitive targets (*M* = 1724.84, *SE* = 73.90), *F*(1, 28) = 24.09, *p* < .001, η_p_^2^ = .46.

*Non-alcoholic v Non-appetitive target (Distractor = Alcoholic)*

As in the coloured version, there was a significant main effect of target type, with faster responses to non-alcoholic targets (*M* = 1567.17, *SE* = 85.22) compared to non-appetitive targets (*M* = 2038.03, *SE* = 91.83), *F*(1, 27) = 35.05, *p* < .001, η_p_^2^ = .57. There was also a significant two-way interaction between target presence and target type, *F*(1, 27) = 6.49, *p* = .017, η_p_^2^ = .19. Simple main effects indicate that the interaction is influenced by the difference in RT between non-alcoholic and non-appetitive targets being greater when the target is absent, compared to when it is present (all *p*’s < .05, and in same direction as main effects).

**Proportional Dwell Time**

*Alcoholic v Non-Alcoholic target (Distractor = Non-appetitive)*

As per the colour version, there was no significant main effect of target type on dwell time (*p* = .11), and no significant interactions between target type, target presence and array size (all interactions *p* > .05).

*Alcoholic v Non-appetitive target (Distractor = Non-alcoholic)*

Consistent with the colour version, there was no significant main effect of target type (*p* = .05). However, although the colour version resulted in a two-way interaction between target presence and target type, this interaction was not present in the greyscale version (*p* > .05).

*Non-alcoholic v Non-appetitive target (Distractor = Alcoholic)*

There was a significant main effect of target type (*F*(1, 27) = 4.64, *p* < .05, η_p_^2^ = .15), with significantly lower dwell time on non-alcoholic stimuli (*M* = .27, *SE* = .01) compared to non-appetitive stimuli (*M* = .31, *SE* = .01), *p* < .05. Mirroring the findings from the colour version, there was a two-way interaction between target type and target presence (*F*(1, 27) = 11.16, *p* < .01, η_p_^2^ = .29), but also between target presence and target type (*F*(1, 27) = 11.16, *p* < .01, η_p_^2^ = .29) and array size and target type (*F*(1, 27) = 10.13, *p* < .01, η_p_^2^ = .27). These were examined further using simple main effects. For non-appetitive stimuli only, lower dwell times were shown when the target was absent (*M* = .24, *SE* = .01) compared to present (*M* = .38, *SE* = .02) *p* < .01. Dwell times were also lower for non-alcoholic stimuli (*M* = .28, *SE* = .02) compared to non-appetitive stimuli (*M* = .38, *SE* = .02) when the target was present, *p* < .01. This is opposite to the coloured version, whereby dwell times were lower for non-alcoholic compared to non-appetitive for *absent* trials. This is likely explained by the greater complexity of greyscale stimuli (i.e., takes longer to detect whether greyscale stimuli is actually present). In addition, dwell times were significantly lower for non-alcoholic stimuli (*M* = .19, *SE* = .02) compared to non-appetitive stimuli (*M* = .30, *SE* = .02) in the small array, *p* < .01. Dwell times were also significantly lower for non-alcoholic stimuli in the small array (*M* = .19, *SE* = .02 compared to the large array (*M* = .35, *SE* = .02), *p* < .01. In sum, these findings are similar to the coloured version in that dwell times are shorted for non-alcoholic compared to non-appetitive stimuli, but for the coloured version this affects trials in which the target is absent, whereas for the greyscale version this affects trials in which the target is present.
